# Correction: The potential of paper-based diagnostics to meet the ASSURED criteria

**DOI:** 10.1039/c8ra90082e

**Published:** 2018-11-12

**Authors:** Suzanne Smith, Jan G. Korvink, Dario Mager, Kevin Land

**Affiliations:** Council for Scientific and Industrial Reasearch (CSIR) Pretoria South Africa ssmith@csir.co.za +27 12 841 3101; Karlsruhe Institute of Technology (KIT) Karlsruhe Germany

## Abstract

Correction for ‘The potential of paper-based diagnostics to meet the ASSURED criteria’ by Suzanne Smith *et al.*, *RSC Adv.*, 2018, **8**, 34012–34034.

For this paper the corresponding author should be as indicated above. Additionally in the Acknowledgements section the following should have been included: We acknowledge support by Deutsche Forschungsgemeinschaft and Open Access Publishing Fund of Karlsruhe Institute of Technology.

The Royal Society of Chemistry apologises for these errors and any consequent inconvenience to authors and readers.

## Supplementary Material

